# Operation status, quality, and premature birth information in obstetrics and gynecology facility websites in Korea: an evaluation study

**DOI:** 10.4069/whn.2025.08.20.1

**Published:** 2025-09-30

**Authors:** Sun-Hee Kim

**Affiliations:** College of Nursing, Research Institute of Nursing Science, Daegu Catholic University, Daegu, Korea

**Keywords:** Access to information, Health facilities, Internet, Premature birth, Quality control

## Abstract

**Purpose:**

This study aimed to identify the operational status and evaluate the quality of websites run by obstetrics and gynecology medical facilities in Korea, as well as to examine the information provided about premature birth.

**Methods:**

Data were collected in April 2022 using the Health Insurance Review and Assessment Service website. A list of tertiary hospitals, general hospitals, hospital (primary-level institutions with ≥30 beds), and midwifery centers, along with their website addresses, was obtained. A total of 566 websites were analyzed. Information on premature birth was retrieved using integrated search functions on medical facility websites. Text- and video-based content related to premature birth was collected and analyzed using NVivo software (Lumivero).

**Results:**

Of 614 obstetrics and gynecology medical facilities, 566 (92.2%) operated websites and 166 (29.3%) had an integrated search function. Premature birth-related information was overwhelmingly text-based (98.7%). The information provided primarily concerned primary prevention. The mean (standard deviation) quality scores of the websites were as follows: purposefulness 4.00 (0.00), credibility 3.58 (0.25), interactivity 3.37 (0.93), currency 3.90 (0.27), ease of use 3.75 (0.18), functionality 4.00 (0.03), design 3.65 (0.38), and confidentiality 3.98 (0.24).

**Conclusion:**

The study revealed disparities in website operation across medical institution classifications, limitations in the format of premature birth information, imbalances in content, and gaps in website quality. Future efforts should focus on diversifying both the format and scope of premature birth information while improving website quality across all levels of facilities, including general hospitals and midwifery centers.

## Introduction

Premature birth, defined as delivery before 37 weeks of gestation, is a critical global health issue that profoundly affects neonatal health and survival [1]. It is the leading cause of neonatal mortality and increases the risk of respiratory, neurological, gastrointestinal, visual, and auditory disorders in surviving infants. Long-term complications include developmental delays, behavioral and social difficulties, emotional problems, and learning disabilities, all of which impose substantial healthcare costs and economic burdens on families and society [2]. Thus, the prevention and management of premature birth continue to receive emphasis, underscoring the importance of providing reliable and accurate access to information for addressing this critical medical condition.

Access to valid and credible health information via the internet significantly influences health awareness, behavior, and clinical outcomes [3], and is an important consideration when developing strategies to address health disparities [4]. Improved access can help women better understand the risk factors associated with premature birth, gain awareness of their own health needs, and evaluate available options, thereby enhancing their ability to make informed health decisions [3,5]. Health information seekers turn to websites to obtain detailed explanations, fill knowledge gaps, or validate their existing understanding of conditions and treatment options [6]. Healthcare professionals and medical information departments rely on extensive resources and digital technologies to help patients understand their conditions and treatments, as well as to overcome barriers in the healthcare process [4]. Medical facility websites, in particular, serve as essential platforms enabling patients and families to access health information and make informed choices. The knowledge acquired can also facilitate better communication and stronger relationships with healthcare providers [6].

During the coronavirus disease 2019 (COVID-19) pandemic, from March 2020 to May 2023 [7], seeking online health information became increasingly common among women, with the majority relying on digital resources for pregnancy-related information [8-10]. The need for social connection, combined with reduced access to in-person prenatal care, made online information seeking a crucial alternative compared with the pre-pandemic period [9]. Dissemination of pregnancy-related health information through digital platforms has been reported as both well-received and potentially effective [11].

Women in particular reported the greatest trust in online information managed by health professionals. However, studies have shown that the quality of online pregnancy-related information varies widely [12,13], with differences depending on the source [12-14]. Furthermore, some websites disseminate misinformation [13,15], raising concerns about potential irreversible societal consequences [16]. Consequently, many women have expressed the need for high-quality online health resources [15]. Identifying the status, content, and quality of such information during the COVID-19 pandemic can help professionals recognize gaps in available resources. In addition, understanding the current state of online health information is essential for promoting the use of reliable resources, improving health literacy, supporting healthy behaviors, and ultimately contributing to better health outcomes. Evaluating and improving the information provided by medical institutions can also help promote health equity.

Despite the importance of ensuring access to high-quality health information, few studies have evaluated the quality of pregnancy-related online resources, and those available have largely focused on preterm premature rupture of membranes [17], obesity during pregnancy [12], and labor induction [13]. For serious conditions such as premature birth, the availability of accurate and accessible information is particularly vital. Nevertheless, research evaluating the content and quality of such information remains insufficient. Therefore, the present study aimed to assess the operational status and quality of websites run by obstetric and gynecological medical facilities in South Korea, with particular focus on premature birth-related content, and to provide recommendations for expanding access and improving quality. The findings of this study can also serve as foundational data for user-centered website design.

The specific objectives of this study are as follows: first, to examine the current operational status of obstetric and gynecological medical facility websites in Korea; second, to identify the formats and contents of premature birth-related information extracted from these websites; and third, to evaluate the quality of these websites.

## Methods

### Study design

This evaluative study employed a descriptive survey to examine the operational status of obstetric and gynecological medical facility websites in South Korea and to assess the quality of the premature birth-related information they provide.

### Study sample

The study targeted websites administered by obstetrics and gynecology medical facilities in South Korea. Website selection was conducted using the “Find Hospitals and Pharmacies” tool available on the Health Insurance Review and Assessment Service website [18]. For this study, medical facilities were classified into four categories: tertiary hospitals, general hospitals, hospital (defined in South Korea as a primary-level medical institution with at least 30 beds, different from general and tertiary hospitals), and midwifery centers [19].

### Data collection

From April 18 to April 31, 2022, the names of medical facilities, their classifications, and corresponding web addresses were collected. For URLs that were incorrect or inaccessible, supplementary searches were performed using Google (google.com) and Naver (naver.com) with the facility names to obtain the correct information.

A total of 1,925 medical facilities were initially identified. General hospitals (n=1) and hospitals (n=1,310) that had ceased operations or lacked active websites were excluded. In two cases, hospitals operated by the same foundation (e.g., a main hospital and its branch) shared the same official website and were therefore counted as a single entry in the analysis. Consequently, 612 medical facilities—including tertiary hospitals, general hospitals, hospitals, and midwifery centers—were examined. Of these, 46 without active websites were excluded from the quality evaluation. Ultimately, 566 websites were included in the analysis.

**Figure 1.**
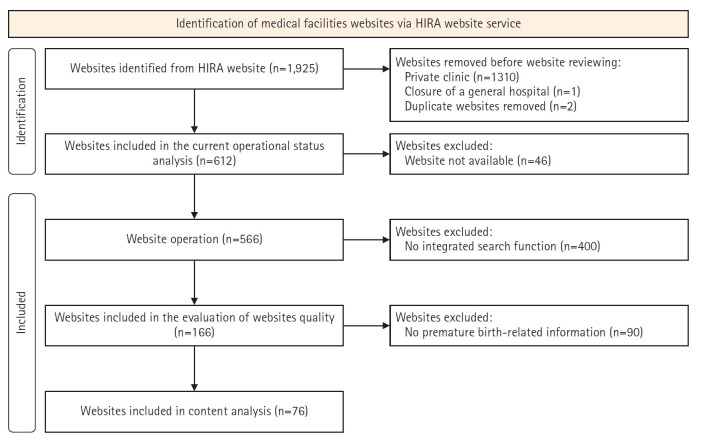
Flow diagram of website selection and evaluation. HIRA: Health Insurance Review and Assessment Service.

To identify premature birth-related information, the integrated search function of each medical facility’s website was used when available. Of the 566 websites, 166 offered this function. The following keywords were employed: “premature birth,” “preterm labor,” “pregnancy,” and “high-risk pregnancy.” Among the 166 websites with integrated search functionality, 76 provided relevant information, while the remaining 90 offered none. Of the 76 websites with information, 28 belonged to tertiary hospitals, 37 to general hospitals, and 11 to hospitals. No relevant information was found on midwifery center websites (Figure 1).

Text-based information was collected as PDF files using NCapture, an NVivo software extension (ver. 12 for Windows; Lumivero, Denver, CO, USA). Video-based information was independently reviewed by two research assistants to extract relevant content. In cases where multiple hospitals under the same foundation shared identical material, only data from the higher-tier hospital were included, and duplicates from lower-tier hospitals were excluded. After removing 53 instances of duplicated text data and four instances of duplicated video data, a total of 320 files were retained for the final content analysis.

### Measurement

#### Website quality

Website quality was evaluated using an assessment tool originally developed by Kang et al. [20], later revised by Choi [21], and subsequently modified by Je and Choi [22]. This tool assessed 20 items grouped into eight subcategories: purposefulness (four items), credibility (three items), interactivity (two items), currency (two items), ease of use (three items), functionality (two items), design (three items), and confidentiality (one item). Each item was rated on a 4-point Likert scale ranging from “strongly disagree” (1 point) to “strongly agree” (4 points), yielding a possible total score of 20–80 points. Higher scores indicated better website quality.

Two research assistants assessed the websites using consistent evaluation criteria. Inter-rater reliability was tested by jointly evaluating 20 identical websites, with discrepancies resolved through discussion to ensure uniform scoring. After reliability was confirmed, the assistants independently assessed the remaining websites. If the evaluators’ scores differed by ≥2 points, a third researcher independently reviewed the website. Discrepancies were then discussed until consensus was reached. The final score for each website was determined by averaging the evaluators’ ratings. The inter-rater reliability for the website quality scores was calculated using the intraclass correlation coefficient, which demonstrated very high reliability (0.99, *p*<.001).

### Data analysis

Collected data were analyzed using IBM SPSS Statistics for Windows, ver. 25.0 (IBM Corp., Armonk, NY, USA). Operational status and information format (text-based or video-based) were analyzed using frequencies and percentages, whereas website quality was analyzed using means and standard deviations (SD). The Kolmogorov-Smirnov test was applied to assess the normality of the distributions, which indicated that website quality data were not normally distributed. Therefore, differences in quality by facility classification were analyzed using the Kruskal-Wallis test, with Mann-Whitney U-tests used for post hoc comparisons.

The content of premature birth-related information was analyzed using NVivo. Directed content analysis [23], based on pre-existing theories or frameworks, was applied to the qualitative data. Information was coded, grouped into themes, and categorized into higher-level codes. These codes were then classified according to the primary, secondary, and tertiary prevention framework developed by Kim et al. [24]. Primary prevention included information on preventing or delaying premature birth, with seven subcategories. Secondary prevention focused on early detection and treatment of risk factors, with five subcategories. Tertiary prevention addressed risk mitigation after premature birth, prevention of complications and disabilities, and facilitation of rehabilitation, with four subcategories [24].

## Results

### Current operational status of obstetric and gynecological medical facility websites in Korea

Of the 612 Korean obstetric and gynecological medical facilities identified, 566 (92.5%) operated websites; among these, 166 (29.3%) offered integrated search functionality. Among the 43 facilities (7.0%) classified as tertiary hospitals, all (100%) maintained websites, and 34 of these (79.1%) included integrated search functions. Of the 282 facilities (46.1%) classified as general hospitals, 280 (99.3%) operated websites, of which 109 (38.9%) supported integrated searches. Among the 271 facilities (44.3%) classified as hospitals, 234 (86.3%) had websites, but only 31 (13.2%) provided integrated search functionality. Finally, of the 16 midwifery centers (2.6%), nine (56.2%) operated websites, but none offered integrated search functionality. These findings are summarized in Table 1.

### Formats of premature birth-related information

Premature birth-related information was identified on 76 websites: 28 from tertiary hospitals, 37 from general hospitals, and 11 from hospitals. Across these, 380 textual information items and five unique videos were collected after excluding four duplicates. Tertiary hospitals provided 131 textual items and four videos. General hospitals offered 216 textual items and one unique video, following the exclusion of four duplicates. Hospitals presented 33 textual items but no videos. Midwifery centers provided no premature birth-related information in any format. A summary of these results is presented in Table 2.

### Content analysis of premature birth-related information extracted from websites

The content analysis of premature birth-related information identified 222 codes for primary prevention, 30 for secondary prevention, and seven for tertiary prevention. Within primary prevention, subcategories included general understanding of premature birth (two themes, 136 codes), preconception health management (4 themes, 14 codes), and prenatal health management during pregnancy (eight themes, 72 codes). Themes covered risk factors and causes of premature birth; infant viability and health problems by gestational week; pregnancy planning and contraception; preconception immune status and chronic disease management; nutritional management; and lifestyle management before conception. For secondary prevention, subcategories included prenatal care for high-risk pregnancies (two themes, 21 codes) and recognition and management of premature birth-related symptoms (two themes, nine codes). Themes included prenatal care planning for high-risk women, early diagnosis and treatment, self-monitoring of premature birth symptoms, and emergency response methods. For tertiary prevention, subcategories included admission and treatment in intensive care units for high-risk pregnant women (two themes, six codes) and postpartum complications (one theme, one code). Themes encompassed diagnostic procedures during hospitalization, inpatient treatment and nursing care, and persistence of postpartum diseases. The content analysis results according to facility classifications are presented in Table 3.

### Website quality evaluation

The mean (SD) scores for the website quality evaluation were as follows: purposefulness, 4.00 (0.00); credibility, 3.58 (0.25); interactivity, 3.37 (0.93); currency, 3.90 (0.27); ease of use, 3.75 (0.18); functionality, 4.00 (0.03); design, 3.65 (0.38); and confidentiality, 3.98 (0.24). The overall mean score across all items was 3.77 (0.16). By facility type, the mean scores were 3.86 (0.10) for tertiary hospitals, 3.82 (0.11) for general hospitals, 3.70 (0.18) for hospitals, and 3.56 (0.13) for midwifery centers. A comprehensive summary of quality evaluation scores by facility category is provided in Table 4. The total website quality score showed a statistically significant difference among facility categories (*p*<.001), ranking as follows: tertiary hospitals, general hospitals, hospitals, and midwifery centers.

## Discussion

This study examined the operational status of obstetric and gynecological medical facility websites in South Korea, as well as the content, format, and quality of premature birth-related information as sources of professional health guidance. The findings revealed disparities in website operation across different medical facility categories, limitations in the formats used to deliver premature birth information, imbalances in the content of prevention categories, and gaps in website quality evaluation.

Disparities in website operation were evident among obstetric and gynecological medical facilities. Although the vast majority of institutions operated websites (92.2%), only 29.3% offered integrated search functionality. Nearly half of midwifery centers had no website, and none provided integrated search functions. These findings align with prior research showing that fewer than two-thirds of websites offered integrated search functions for hypertension-related information [25]. Integrated search functionality is critical for meeting user needs by enabling efficient and convenient retrieval of electronic information [26]. In this study, most tertiary hospitals provided such functionality, whereas adoption was markedly lower among secondary and primary care institutions, including general hospitals, hospitals, and midwifery centers. These results underscore the discrepancies in information accessibility across facility types. Because primary care institutions are the first point of contact for community health and provide essential information during emergencies such as preterm birth, improving digital accessibility is strongly recommended. Strategies should include strengthening search functions and information architecture, as well as promoting public–private partnerships to integrate reliable health information into primary care websites through API-based connections with national health information portals.

Premature birth-related information on institutional websites was presented predominantly in text format, with only a small number providing video content. This finding is consistent with earlier research showing limited diversity in the formats used to deliver hypertension-related information online [25]. According to a systematic review, maternal health information is most commonly provided online in text-and-image formats [27]. However, internet users seeking health information often prefer general or nonportal platforms over institutional websites due to greater accessibility, convenience, and more frequent updates [28]. For example, YouTube (Google LLC, Mountain View, CA, USA)—used by 79.9% of internet users in South Korea for video-based information searches—has become one of the most popular platforms [28]. Among information formats associated with improved patient outcomes, video demonstrated the highest proportion of positive effects compared to other formats [27]. To address current limitations, institutional medical websites must diversify the delivery formats of health information. One strategy could involve integrating audiovisual media, such as YouTube content, into medical websites to enhance the delivery of premature birth-related information. However, this must be approached cautiously, given the variability in accuracy and reliability of YouTube content [29]. Interpreting internet-based health information can be difficult for users due to complexity, varying levels of health literacy, and the risk of misinterpreting one’s health status based on information intended for others [6]. The use of conversational artificial intelligence (AI) tools, such as ChatGPT (OpenAI, San Francisco, CA, USA), may play an important role in disseminating and acquiring health information. Currently, only 17.8% of domestic internet users report using such tools, with most relying on them for learning and knowledge acquisition [28]. While conversational AI technology has limitations in terms of accuracy and reliability, it is highly valued for its accessibility, rapid responses, interactivity, and convenience [28,30]. Given that this study’s website quality evaluation revealed relatively low interactivity scores, it is clear that the information provided did not meet the expectations of health information seekers and was insufficient for optimizing health outcomes. Incorporating interactive functions or conversational AI technologies will therefore be critical to improving website quality. Health professionals, as guardians and distributors of reliable health information amidst a “sea of misinformation” [6,31], must account for multiple factors, including user preferences, levels of health literacy, and the demand for interactivity. Efforts should prioritize enhancing the dissemination of health information on institutional medical websites by integrating features that balance accuracy, trustworthiness, and effective communication with interactive elements.

In terms of content, the premature birth-related information provided on medical facility websites was largely concentrated on primary prevention strategies, particularly general information about premature birth and prenatal health management during pregnancy. In contrast, content related to secondary and tertiary prevention was markedly limited. For secondary prevention, only minimal details were offered regarding prenatal care for high-risk pregnancies and the recognition and management of premature birth symptoms. Tertiary prevention information was similarly sparse, including only limited guidance on admission and treatment in intensive care units for high-risk pregnant women and postpartum complications. These findings are consistent with prior studies analyzing YouTube content on premature birth prevention, which concluded that available information focused primarily on risk factors and general overviews of premature birth [29]. This heavy emphasis on primary prevention reflects the limitations of the one-way transmission of online health information. In clinical situations requiring hospital visits or inpatient care, patients often receive information directly from healthcare professionals, which reduces reliance on online resources by both providers and seekers. However, in high-risk premature birth cases or during hospitalization, the limited online availability of detailed information may drive patients to seek clarification and reassurance [6]. While advances in health information technology have improved disease diagnosis, monitoring, self-management, and risk prediction, experts recommend further development of such technologies to bridge information gaps and support individuals with varying levels of health literacy [4,5]. Thus, healthcare professionals should ensure that institutional websites include accurate information on risk management in high-risk contexts and during hospitalization. Expanding secondary and tertiary prevention content will be essential to provide comprehensive resources and enable continuous access to relevant information through open communication channels.

This study also found that primary prevention information concerning prenatal health management was more prevalent during pregnancy than before conception, consistent with previous analyses of YouTube content on premature birth prevention [29]. One study reported that approximately 40% of participants used the internet to obtain information on managing health problems, while about 33% searched for pregnancy complication information. However, an examination of website content revealed a strong emphasis on primary prevention, with minimal attention to high-risk pregnancies and preterm birth [32]. This imbalance highlights a critical gap in the types of information available. Although comprehensive preconception health management plays a vital role in preventing premature births [33], online content disproportionately emphasizes prenatal care. Future website content should therefore include more information on preconception health management. Regarding secondary prevention strategies, this study revealed a relative lack of guidance on prenatal care for high-risk women and the recognition and management of premature birth symptoms. The onset of premature birth symptoms represents a critical period during which self-monitoring and timely emergency management can help secure the necessary window for medical interventions to prevent preterm delivery [34]. In terms of tertiary prevention, the results highlighted the scarcity of information addressing diagnostic procedures for high-risk pregnancies, inpatient treatment for women at elevated risk, and long-term health issues. The absence of tertiary prevention information may hinder effective treatment and management, weaken patient–provider communication, and exacerbate complications, ultimately increasing the risk of premature birth and associated long-term health problems for both mothers and infants [35]. Therefore, websites should expand their coverage of preconception health management as well as secondary and tertiary prevention strategies for premature birth. Such expansion would strengthen patient education, improve access to essential health information, and facilitate the development of more comprehensive care strategies.

The website quality evaluation, based on usability-related criteria, also revealed notable disparities across medical facility categories. Interactivity received the lowest mean score, followed by credibility, design, and ease of use. Websites operated by tertiary hospitals generally provided higher-quality information, whereas those maintained by hospitals and midwifery centers scored lower in credibility, interactivity, and currency. These differences likely reflect variations in staffing expertise, financial and infrastructural resources, and institutional priorities. Specifically, credibility was undermined by the absence of author attribution and failure to list content limitations. Currency scores were reduced because many websites did not display the date of initial content creation. Ease of use was negatively affected by the lack of help guides, while design scores suffered due to poor layout, low-quality visuals, or a complete absence of graphics.

This study did not evaluate the intrinsic quality of content—such as accuracy, evidence-based validity, or fairness—nor the readability of website text, which determines how easily users can understand the information provided. However, many prior studies assessing specific health topics have reported low levels of both quality and readability [36,37]. Certification can serve as an indicator of quality and reliability. For example, the Mayo Clinic, a leading nonprofit medical institution in the United States, has received Health on the Net Code certification, a standard that ensures the quality and reliability of online health information. This certification has contributed to the Mayo Clinic’s health information being ranked second-highest in quality among organizations evaluated in a prior study [38]. Large-scale institutions such as the Mayo Clinic underscore the importance of maintaining high-quality online health information to promote equitable access and facilitate timely connections between patients and healthcare services. To address quality disparities, medical institutions should establish policies for resource allocation and technological upgrades to consistently maintain their websites above a minimum quality standard. Ensuring the availability of high-quality online health information would enhance patients’ ability to access trustworthy sources and guarantee usability for all users, regardless of the size of the institution.

This study also represents an important early effort in pregnancy health and provides practical insights into the operational status and information accessibility of medical facility websites in the post-COVID-19 digital health information environment. First, primary care facilities such as hospitals and midwifery centers—typically used during the early stages of pregnancy—generally exhibited poor website management. Even when websites were available, their usability was often limited. Second, while hospital websites tended to provide relatively reliable health information, they lacked user-friendly features such as integrated search functions, which restricted usability and may have contributed to low utilization rates. By contrast, commercial websites often delivered less accurate and less trustworthy information [9], creating disparities in access that could hinder pregnant women from obtaining appropriate health resources. Third, with regard to information format, most content was confined to basic text-and-image presentations rather than videos, despite the latter being more effective in promoting health behavior change. As a result, institutional websites lacked personalized information delivery and interactive features for users. Fourth, there was a substantial deficiency in information related to the prevention and management of preterm birth, underscoring the need for improved content development in this area. Overall, the study highlights the structural limitations of medical institution websites and emphasizes the need for quality improvements to enhance accessibility and usability for pregnant women. Specifically, the findings suggest that websites managed by healthcare professionals should refine their information architecture and provide balanced, evidence-based pregnancy content—including preterm birth information—to ensure both accuracy and reliability.

While this study made important contributions by identifying the operational status, quality, and limitations of premature birth-related information on medical facility websites, several limitations must be acknowledged. First, it did not assess the actual needs, usage patterns, or satisfaction of users, making it difficult to evaluate the effectiveness of the information provided. Future research should therefore include user-centered qualitative studies and detailed analyses of digital resource utilization. Second, the study did not assess the intrinsic quality or readability of the website content. Future studies should address these aspects to inform the development of high-quality websites for women. Third, the analysis was limited to information on institutional websites and did not consider content available on other digital platforms. It is possible that secondary and tertiary prevention information related to premature birth risk, diagnosis, management, and inpatient care was provided elsewhere, but integrated information platforms were not examined. Fourth, because integrated search functions rely on pre-defined keywords [26], the terms used in this study may not have matched the websites’ keyword systems, potentially leading to omissions. Thus, the findings should be interpreted cautiously. Fifth, although this study analyzed text- and video-based formats, it did not examine visual elements such as graphic design or interface usability, which can substantially affect how information is delivered and understood. These limitations should be considered when interpreting the results.

In conclusion, although most obstetric and gynecological medical facilities in South Korea operated websites, integrated search functions and visual formats such as videos were largely absent. Midwifery centers, in particular, lacked integrated search functionality altogether, revealing significant disparities in information provision. Premature birth-related content was heavily concentrated on primary prevention—such as general knowledge and prenatal care—while secondary and tertiary prevention content was insufficient, limiting the comprehensiveness of available resources. Therefore, users may need guidance toward reliable supplementary platforms, such as YouTube or mobile applications, to access more complete information and improve communication with healthcare professionals.

The websites of tertiary hospitals generally provided higher-quality information compared with those of hospitals and midwifery centers, which scored lower in credibility, interactivity, currency, ease of use, and design, demonstrating quality gaps across facility types. Efforts should focus on diversifying both the content and formats of premature birth-related information, while enhancing the credibility, timeliness, interactivity, and user-friendliness of websites, especially in hospitals and midwifery centers.

Future research should conduct in-depth evaluations of user needs, usage behaviors, and satisfaction levels, along with systematic assessments of content quality and readability. Broader analyses of premature birth-related information across multiple digital platforms, including YouTube and mobile applications, are also needed. Finally, future studies should examine the impact of visual and design elements on a website’s ability to effectively convey health information.

## Figures and Tables

**Table 1.** Current operational status of obstetrics and gynecology medical facility websites in South Korea (N=612)

Characteristic	Categories	n (%)				
		Total	Tertiary hospital	General hospital	Hospital	Midwifery center
Medical facility	-	612 (100)	43 (7.0)	282 (46.1)	271 (44.3)	16 (2.6)
Website operation (n=612)	Yes	566 (92.5)	43 (100)	280 (99.3)	234 (86.3)	9 (56.2)
	No	46 (7.5)	0 (0)	2 (0.7)	37 (13.7)	7 (43.8)
Integrated search operation (n=566)	Yes	166 (29.3)	34 (79.1)	109 (38.9)	31 (13.2)	0 (0)
	No	400 (70.7)	9 (20.9)	171 (61.1)	203 (86.8)	9 (100)

**Table 2.** Format of premature birth information by facility type (76 websites) (N=385)

Facility type	Format of premature birth information, n (%)	
	Text	Video
Total	380 (98.7)	5 (1.3)^†^
Tertiary hospital	131 (97.0)	4 (3.0)
General hospital	216 (99.5)	1 (0.5)
Hospital	33 (100)	0 (0)
Midwifery center	0 (0)	0 (0)

† The same video from one tertiary hospital and four general hospitals that were part of a medical center is counted as one video from a tertiary care hospital.

**Table 3.** Content analysis of premature birth-related information extracted from websites (76 websites)

Categories	Themes	Total	Tertiary hospital	General hospital	Hospital	Midwifery center
Primary prevention		131	222	79	12	0
General understanding of premature birth		80	136	55	1	0
	Risk factors and causes of premature birth	128	77	50	1	0
	Baby's viability and health problems by the week of gestation	8	3	5	0	0
Preconception health management		6	14	7	1	0
	Pregnancy planning and contraception	5	3	1	1	0
	Preconception immune status assessment and chronic disease management	5	2	3	0	0
	Preconception nutritional management	2	0	2	0	0
	Preconception lifestyle management	2	1	1	0	0
Prenatal health management during pregnancy		45	72	17	10	0
	Hospital selection	3	2	1	0	0
	Chronic disease management during pregnancy	7	5	2	0	0
	Management of pregnancy-related diseases	18	14	4	0	0
	Fetal anomaly testing	9	3	1	5	0
	Nutritional management during pregnancy	16	11	4	1	0
	Prevention of epidemic infections during pregnancy	3	1	0	2	0
	Lifestyle management during pregnancy	13	9	3	1	0
	Stress and emotional management during pregnancy	3	0	2	1	0
Secondary prevention		16	30	14	0	0
Prenatal care for high-risk pregnancies		13	21	8	0	0
	Prenatal care planning for high-risk women with premature birth risk	10	5	5	0	0
	Early diagnosis and treatment for high-risk women with premature birth risk	11	8	3	0	0
Recognition and management of premature birth symptoms		3	9	6	0	0
	Self-monitoring of premature birth risk symptoms	8	4	4	0	0
	Emergency response methods for detecting premature birth risk	3	0	3	0	0
Tertiary prevention		3	7	2	2	0
Admission and treatment in intensive care units for high-risk pregnant women		2	6	2	2	0
	Diagnosis and diagnostic procedures during hospitalization	1	0	0	1	0
	Inpatient treatment and nursing care	5	2	2	1	0
Postpartum complications		1	1	0	0	0
	Persistence of postpartum disease	1	1	0	0	0

**Table 4.** Website quality evaluation (N=566)

Categories	Items	Mean±SD					Kruskal-Wallis H	*p* (Mann-Whitney U, post-hoc)
		Total	Tertiary hospital, a	General hospital, b	Hospital, c	Midwifery centers, d		
Purposefulness	Subtotal	4.00±0.00	4.00±0.00	4.00±0.00	4.00±0.00	4.00±0.00	0.00	>.999
	Purpose statement	4.00±0.00	4.00±0.00	4.00±0.00	4.00±0.00	4.00±0.00		
	Target audience specification	4.00±0.00	4.00±0.00	4.00±0.00	4.00±0.00	4.00±0.00		
	Site name	4.00±0.00	4.00±0.00	4.00±0.00	4.00±0.00	4.00±0.00		
	URL, domain	4.00±0.00	4.00±0.00	4.00±0.00	4.00±0.00	4.00±0.00		
Credibility	Subtotal	3.58±0.25	3.83±0.24	3.64±0.08	3.47±0.31	3.30±0.30	140.95	<.001 (a>b>c>d)
	Author attribution	3.76±0.51	3.76±0.44	3.93±0.25	3.58±0.64	3.11±0.55		
	Content expertise	3.93±0.25	4.00±0.00	4.00±0.00	3.84±0.37	3.78±0.44		
	Specification of information limitations	3.05±0.24	3.72±0.45	3.00±0.00	3.00±0.10	3.00±0.00		
Interactivity	Subtotal	3.37±0.93	3.80±0.43	3.27±0.92	3.40±0.98	3.69±0.92	48.32	<.001 (a>b>c, d>b, d>c)
	Feedback collection mechanisms	3.56±0.95	3.81±0.41	3.57±0.99	3.49±0.97	3.72±0.83		
	Handling of user opinions	3.18±0.99	3.78±0.47	2.97±0.93	3.31±1.06	3.67±1.00		
Currency	Subtotal	3.90±0.27	3.89±0.23	3.99±0.05	3.83±0.35	3.03±0.26	125.44	<.001 (a>c>d, b>c, b>d)
	Initial creation date indication	3.82±0.51	3.78±0.47	3.99±0.10	3.68±0.65	2.17±0.35		
	Linked sites	3.99±0.13	4.00±0.00	4.00±0.00	3.97±0.19	3.89±0.33		
Ease of use	Subtotal	3.75±0.18	3.93±0.14	3.80±0.16	3.67±0.16	3.33±0.25	133.86	<.001 (a>b>c>d)
	Site map	3.98±0.14	4.00±0.00	4.00±0.00	3.97±0.17	3.56±0.53		
	Usage guidelines/help	3.28±0.50	3.79±0.41	3.41±0.49	3.06±0.38	2.44±0.46		
	Location display	3.99±0.10	3.99±0.08	4.00±0.06	3.98±0.13	4.00±0.00		
Functionality	Subtotal	4.00±0.03	3.99±0.04	4.00±0.00	4.00±0.05	4.00±0.00	4.61	.203
	Accessibility of materials	4.00±0.06	3.99±0.08	4.00±0.00	3.99±0.09	4.00±0.00		
	Cost of accessing information	4.00±0.00	4.00±0.00	4.00±0.00	4.00±0.00	4.00±0.00		
Design	Subtotal	3.65±0.38	3.50±0.39	3.87±0.20	3.42±0.37	3.28±0.58	205.26	<.001 (b>a>c, d)
	Expressiveness	3.74±0.45	3.35±0.57	3.92±0.29	3.61±0.49	3.33±0.66		
	Layout	3.70±0.48	3.85±0.35	3.93±0.26	3.43±0.54	3.33±0.50		
	Graphics	3.49±0.54	3.30±0.51	3.76±0.42	3.22±0.50	3.17±0.71		
Confidentiality	Privacy protection measures	3.98±0.24	4.00±0.00	4.00±0.00	3.94±0.37	4.00±0.00	8.59	.035 (a, b>c)
Total		3.77±0.16	3.86±0.10	3.82±0.11	3.70±0.18	3.56±0.13	113.50	<.001 (a>b>c>d)
